# Dr. William H. Wathen, LL.D.

**Published:** 1913-12

**Authors:** 


Dr. William H. Wathen, LL.D., of Louisville, Ky., a graduate of the University of Louisville, Medical Department, in 1870, a member of the American Gynecological Society, one of the founders and for many years dean of the Kentucky School of Medicine, which, in 1908, was merged with the Medical Department of the University of Louisville, and since that time professor of abdominal surgery and gynecology in the latter institution, died at St. Anthony's Hospital, Louisville, after a short illness, on October 7, aged 67 years.

